# 662. I*n vitro* antibacterial activity of contezolid compared to linezolid and vancomycin in clinical isolates from China

**DOI:** 10.1093/ofid/ofac492.714

**Published:** 2022-12-15

**Authors:** Shanmei Wang, Hao Li, Jianjian Cheng

**Affiliations:** Henan Province People’s Hospital, Zhengzhou, Henan, China; Queshan People's Hospital, Zhumadian, Henan, China; Henan Province People’s Hospital, Zhengzhou, Henan, China

## Abstract

**Background:**

Linezolid and vancomycin are insufficient in the treatment of drug-resistant Gram-positive bacteria due to adverse drug reactions and drug resistance, and new antibiotics are needed, especially for patients with myelosuppression and vancomycin-resistant *Enteroccocus* (VRE) infection. Contezolid (CZD) is a novel oral oxazolidinone with potent activity against Gram-positive pathogens, including methicillin-resistant *Staphylococcus aureus* (MRSA) and VRE. We evaluate the *in vitro* antibacterial activity of contezolid compared to linezolid and vancomycin in Gram-positive clinical isolates.

**Methods:**

176 clinical isolates were obtained from 176 sterile body fluids from patients at 3 tertiary care hospitals in China, and the minimal inhibitory concentrations (MICs) of contezolid, linezolid and vancomycin determined by broth microdilution assay.

**Results:**

Contezolid exhibits excellent antibacterial activity against prevalent Gram-positive bacteria, *Staphylococcu*s spp. and *Enterococcus* spp., including methicillin-resistant *Staphylococcus aureus* (MRSA), methicillin-resistant and coagulase-negative *Staphylococcus* (MRCNS), and vancomycin-resistant *Enterococcus* (VRE). MIC_90_ values of contezolid were 2 mg/L for all MRSA and VRE isolates and 1 mg/L for MRCNS isolates. The antibacterial activity of contezolid against *Staphylococcus* spp., including *S. aureus*, was essentially similar to linezolid, and slightly higher than or similar to vancomycin. Antibacterial activity of contezolid against *Enterococcus*, including VRE, was similar to linezolid, while being slightly lower than for vancomycin in vancomycin-susceptible isolates. Consistent with the shared antibacterial mode of action, bacterial strains resistant to linezolid were cross-resistant to contezolid.

**Table 1.**
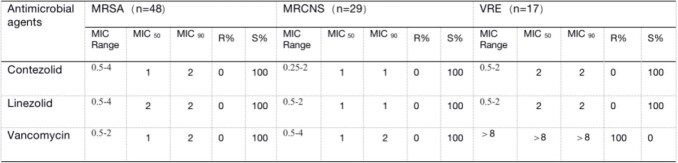
Antimicrobial susceptibility testing results of clinical bacterial strains (MICs, mg/L)

**Conclusion:**

Contezolid, a novel oxazolidinone drug, exhibits potent *in vitro* antibacterial activity against clinical isolates of common Gram-positive pathogens, including multidrug-resistant (MDR) Gram-positive bacteria, MRSA, MRCNS, and VRE. Contezolid has antibacterial activity similar to standard-of-care antibiotics linezolid and vancomycin. Results suggest that contezolid can play an important role in treatment of MDR Gram-positive bacterial infections.

**Figure 1.**
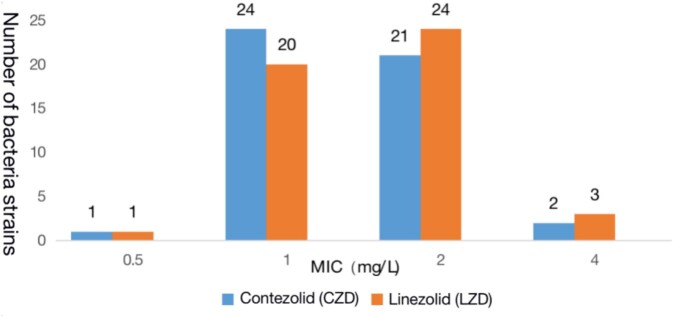
Distribution of MICs of CZD or LZD against 48 strains of MRSA

**Figure 2.**
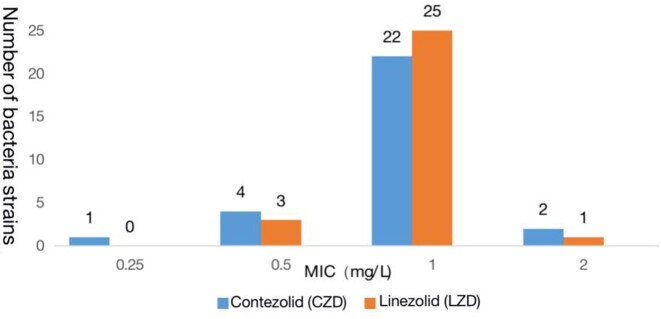
Distribution of MICs of CZD or LZD against 29 strains of MRCNS

**Figure 3.**
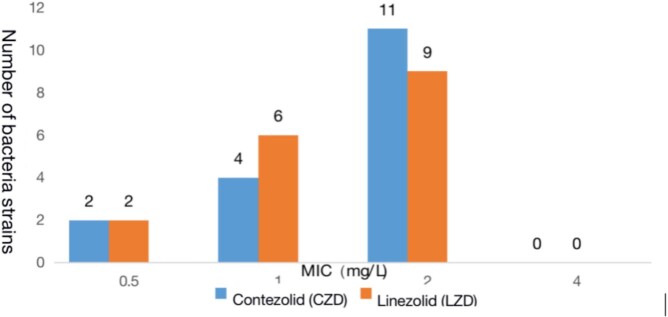
Distribution of MICs of CZD or LZD against 17 strains of VRE

**Disclosures:**

**All Authors**: No reported disclosures.

